# Changes in tobacco imagery and smokers’ depiction in Spanish top-grossing films before and after the implementation of a comprehensive tobacco control policy in Spain

**DOI:** 10.18332/tid/162700

**Published:** 2023-05-19

**Authors:** Ariadna Feliu, Alex Quintero, Armando Peruga, Dolors Carnicer-Pont, Laura Antón, Juan M. Rey-Pino, Esteve Fernández

**Affiliations:** 1 Unidad de Control del Tabaco, Centro Colaborador de la OMS para el Control del Tabaco, Institut Catala d'Oncologia, L'Hospitalet de Llobregat, Barcelona, Espana; 2 Grupo de Investigacion en Control del Tabaco, Institut d'Investigacio Biomedica de Bellvitge-IDIBELL, L'Hospitalet de Llobregat, Barcelona, Espana; 3 Centro de Investigacion Biomedica en Red de Enfermedades Respiratorias, (CIBERES), Madrid, Espana; 4 Centro de Epidemiologia y Politicas de Salud, Facultad de Medicina Clinica Alemana, Universidad del Desarrollo, Santiago, Chile; 5 Departamento de Comercializacion e Investigacion de Mercados, Facultad de Ciencias Economicas y Empresariales, Universidad de Granada, Granada, Espana; 6 Departamento de Ciencias Clinicas, Facultad de Medicina, Universitat de Barcelona, Hospitalet de Llobregat, Barcelona, Espana

**Keywords:** tobacco imagery, smokers’depiction, films, Spain, legislation

## Abstract

**INTRODUCTION:**

As more restrictions on tobacco marketing communication are implemented, tobacco marketing has persisted through smoking in films. Our aims were to assess changes in tobacco imagery exposure in Spanish top-grossing films before and after the banning of tobacco advertising in Spain, and to determine whether the depiction of smoking characters has changed over the years.

**METHODS:**

A repeated cross-sectional study measured the tobacco content in the 10 Spanish top-grossing films in 2005, 2010 and 2015 (n=30) before and after a complete tobacco advertising ban. We conducted a descriptive and regression analysis of changes in tobacco impressions by year.

**RESULTS:**

The 30 films contained 1378 tobacco occurrences (90.2% positive for tobacco) with a median length of eight seconds onscreen. Total tobacco occurrences deemed positive for tobacco interests significantly increased in 2010 and 2015 compared to 2005. However, we observed decreased odds of tobacco brands appearances (OR=0.25; p<0.001) in 2010 and of implied tobacco use (OR=0.44; p=0.002), and tobacco brands appearances (OR=0.36; p<0.001) in 2015 compared to 2005. There was a change of pattern in the type of role smokers played from a leading role to a supporting one (p<0.001). The population reach of positive for tobacco occurrence in Spanish top-grossing films decreased from 15.9 (95% CI: 15.86–15.86) per 1000 spectators in 2005 to 0.8 (95% CI: 0.82–0.82) in 2015.

**CONCLUSIONS:**

The implementation of a ban on complete tobacco product advertising was followed by a decrease in tobacco incidents across top-grossing Spanish films. Yet, exposure to smoking in films is still unacceptably high.

## INTRODUCTION

Tobacco depiction in films has been identified by the World Health Organization (WHO) as an important vehicle for promoting smoking^1^. Exposure to tobacco imagery has been associated with smoking initiation, reinforcing tobacco use among smokers and facilitating the relapse of ex-smokers^2^, thus undermining efforts to decrease smoking prevalence^3^. In Spain, despite the fact that smoking prevalence significantly decreased from 39.8% to 18.7% in adolescents aged 15–24 years from 1997 to 2018, declining trends in tobacco consumption have slowed down since 2011^4^.

Article 13 of the WHO Framework Convention on Tobacco Control (FCTC)^5^ requires Parties to comprehensively ban tobacco advertising, promotion, and sponsorship (TAPS), including restricting TAPS on radio, television (TV), print media and other media including films. In Spain, direct tobacco advertising on TV was first banned in the 1990s (Law 34/1988, Law 25/1994). Yet, it was not after the ratification of the WHO FCTC in 2005 that TAPS were comprehensively banned (Law 28/2005, Law 7/2010) in all media^6^.

Millet and Glantz^3^ suggested that in those countries with stronger restrictions on conventional marketing communication of tobacco products, the relative importance of smoking in films as a stimulant for youth smoking is higher unless onscreen smoking is regulated.

Public health concerns about smoking in films, however, surpass merely the amount of exposure to tobacco impressions. Over decades, tobacco industry media communication has strategically targeted various population sectors based on their sociodemographic characteristics^7^. Marketing seeks to develop and associate images or themes appealing to the target audience with a consumer product^8^. Media campaigns are tailored for these consumer segments by using special models, messages, settings, values, beliefs, and product features^8^ to meet smokers’ specific needs or wants^9^. Although several studies have researched the role of onscreen smokers’ depiction in promoting smoking among youth, little evidence is available on how the features of onscreen smoking has changed in recent years in films in Spain, and elsewhere, to attract different sectors of the population.

Accordingly, our aims were: 1) to describe and assess the changes in exposure to tobacco imagery in Spanish top-grossing films before and after completely banning tobacco products marketing communication in Spain with the implementation of a comprehensive tobacco control policy (Law 28/2005; Law 42/2010, a modification of the previous one); and 2) to determine whether the depiction of smoking characters has changed over the years to further understand if tobacco imagery in films has been designed to target different segments of smokers over time.

## METHODS

### Design

**Figure 1:**
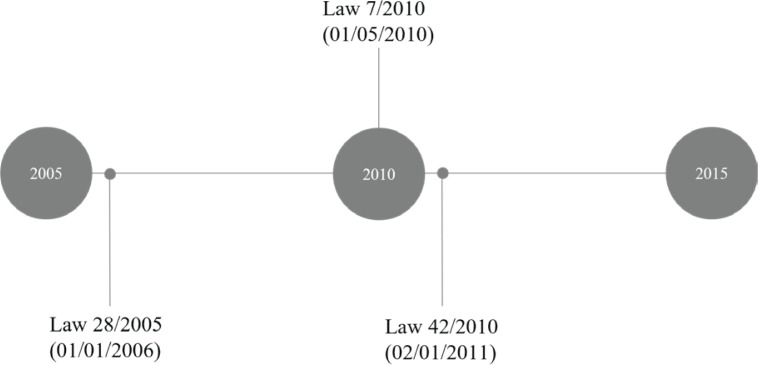
Timeline of the data collection time-point, and the legal framework of tobacco marketing communications ban in Spain in the period 2005–2015

We have conducted a cross-sectional study with three time-points in 2005, 2010 and 2015, before and after the Law 28/2005 (and its amendment, Law 42/2010), which came into force on 1 January 2006 and 2 January 2011, respectively; and the Law 7/2010, which came into force on 1 May 2010 (Figure 1).

Law 28/2005 introduced the prohibition of sponsorship and all types of strategies of marketing communication of tobacco products and the promotion of the referred products in all media and forms. In 2010, an amendment (Law 42/2010) specified that this prohibition included the broadcasting of programs or images on any media. This applied to the presenters, collaborators or guests that are seen smoking, and who mention or show, directly or indirectly, brands, trade names, logos or other symbols identifying or associated with tobacco products. That year, Law 7/2010 on General of Audiovisual Communication, banned all covert commercial communication, including product placement in media. These restrictions should apply to films and TV series, although previously mentioned laws do not specifically prohibit tobacco imagery, since they are commercial communications.

### Sample

We measured the tobacco content of the 10 annual Spanish top-grossing feature films shown in Spain in cinemas for the years 2005, 2010 and 2015 (n=30 films), identified from Institute of Cinematography and Audiovisual Arts (ICAA) annual report^10^ and the Spanish film catalog^11^. The ICAA is an agency attached to the Ministry of Culture of Spain that, among other things, keeps statistics on the viewership of films shown in Spain.

The films included those that were: 1) released in Spanish cinemas in the years under study (2005, 2010 and 2015); and 2) produced with at least 25% funding from Spanish companies. We excluded one animation film for having different features that may make comparison with other films difficult. For each film excluded, we included the next film with the highest audience in the study year that met the inclusion and exclusion criteria. The films included in the sample are indexed in Supplementary file Table S1.

### Codification process

Coding each film began at the start of each film and continued until the end of the credits. All films were coded by two independent trained observers who had a high agreement after piloting codification in 5 films (AF, AQ). Disagreements between both observers were solved in periodic meeting sessions with a third researcher (EF), where clips of the films were watched again, if necessary. Tobacco occurrences were coded into four categories, previously described by Lyons et al.^12^. Following the same methodology, we calculated the items described below.

*Explicit tobacco use*

We coded the use of any tobacco product [cigarette, roll-your-own (RYO), cigar, pipe, waterpipe, or other] or vaporizer by a character. Joints were coded as RYO tobacco, even if their tobacco content could not be ascertained. For each explicit tobacco use, apart from the type of tobacco product used, we coded the character’s role in the film (leading, supporting or extra), sex (men, women), age appearance (minors aged <18 years, young adults aged 18–30 years, and other adults aged >30 years), the smoking policy environment (closed, semi-opened or opened), the type of venue where smoking took place (home, hotel, bar/restaurant, education center, governmental facility, healthcare center, public transport, night club, station/airport, workplace, cultural venues, or other), and the social context for smoking (defiance, social acceptance, smoking policy environment, identity, habit, or pleasure). The classification of social context for smoking is further described in Supplementary file Table S2.

*Implied tobacco use*

Any inference to the action of ‘smoking’ occurring without actual use onscreen (i.e. a gesture or a comment about going for a cigarette). These occurrences were further coded as ‘verbal’ or ‘nonverbal’.

*Tobacco paraphernalia*

Onscreen appearance of unlit tobacco products or tobacco-related materials, coded by type (cigarette, RYO, tobacco packs, matches, lighters, ashtrays, cigarette butt, cigarette case, cut tobacco, cigarette filter or paper, smoking area signs, or other).

*Tobacco brand appearance*

Onscreen presence of clear and unambiguous tobacco branding, even if the name of the brand did not appear, whether sold in Spain or not.

We recorded tobacco occurrences by scene. Occurrences in different coding categories but in the same scene were recorded as two separate ones. Occurrences that crossed a transition from one scene to the next were recorded as two separate ones. Multiple explicit tobacco use occurrences in the same scene by different characters were codified as two different occurrences to be able to individually characterize how each onscreen smoker is depicted. However, multiple occurrences of the same type of paraphernalia, brand or implicit use in the same scene were codified as a single occurrence (i.e. two lighters on a table or two tobacco-related products of the same brand).

Each occurrence was classified as positive, negative, or neutral for tobacco interests, depending on its impact valence towards smoking. A tobacco occurrence was considered positive for tobacco when observers concluded that tobacco was cast as attractive or without consequences for health, negative for tobacco when tobacco was depicted as harmful for health, characters were encouraged to quit, or a smoke-free sign was onscreen, and neutral when an occurrence mixed both a positive and negative valence.

Finally, we also reported two independent variables that were hypothesized to be associated with the frequency of tobacco imagery exposure and could be subject to regulation. These variables were film genre (drama, comedy, thriller/terror or other) and film rating (general audience, not recommended for audiences aged <13 years, or not recommended for audiences aged <16 years). In Spain, there are no explicit restrictions on film rating related to tobacco content. Information on the film genre and rating of each film was obtained from the ICAA Spanish Film Catalogue^11^.

### Outcomes

*Tobacco occurrences*

Total tobacco occurrences in the sample were calculated by summing all four tobacco impressions types across all scenes in the 30 films watched. Similarly, we calculated the duration of exposure to tobacco imagery in seconds by summing the number of seconds an occurrence was on screen.

*Population exposure*

Reach, as defined by Peruga et al.^13^, is the population exposure to the tobacco imagery. It is a measure of frequency of tobacco impressions seen by the audience. Reach was calculated in total and by type of valence of occurrences (positive for tobacco or other) in each year. We multiplied the number of tobacco occurrences in each film by their duration and multiplied the result by the total audience that watched the film each year in movie theaters to obtain the total tobacco person/time impressions per film. Then, we calculated the exposure rate by dividing the sum of all tobacco person/time impressions with a positive valence for tobacco across all 10 films of interest over the person/time duration of the 10 films considered in each year (sum of products of film duration and audience of the ten films in each year).

### Statistical analysis

Coding data from each film were entered directly on a single Microsoft Access (Version 2102) database as the films were watched and discrepancies solved. These databases were merged in a single database once fieldwork was completed. We calculated median and interquartile range (IQR) of the length of the occurrences in seconds by type of occurrence and valence and overall, and compared them by premiere year (2005, 2010 and 2015) using the Kruskal-Wallis test. We also described the characteristics of explicit tobacco use occurrences with frequencies and percentages stratified by their release year in Spain (2005, 2010, 2015) and we used chi-squared test to test differences across variables. We fitted multivariable linear regression models to assess the variables associated with positive for tobacco occurrences. Finally, we obtained the odds ratios (ORs) for changes in impressions by year according to their valence and typology by means of a multinomial logistic regression model, adjusted for occurrence valence (positive for tobacco vs other) and type of occurrence (implied, explicit, paraphernalia, logo, or brand). A p<0.05 was deemed statistically significant. Analyses were conducted using SPSS (Version 25).

## RESULTS

### Tobacco occurrence

The sum of the running time of the 30 films analyzed was 3241 minutes of which 360 (11%) contained at least one tobacco occurrence positive for tobacco. Only two films did not contain any occurrence positive for tobacco (Buried and Extinction). In total, we observed 1269 positive for tobacco occurrences: 41.8% were of explicit tobacco use, 40.2% of tobacco paraphernalia, 9.6% of implicit tobacco use, and 8.4% of tobacco brand appearances. These frequencies stayed stable over the whole period of 10 years with no statistically significant differences across years.

**Figure 2:**
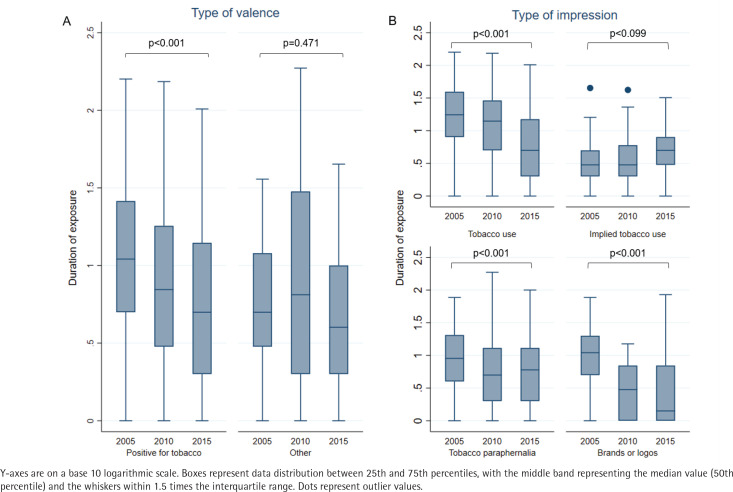
Box-plot of median duration of exposure to occurrences in seconds according to: A) type of valence [positive for tobacco or other (negative or neutral)]; and B) type of impression (tobacco use, implied tobacco use, tobacco paraphernalia or brands or logos) by premiere year in 30 Spanish films

Tobacco occurrences in films in all three points in time analyzed had a median length of 8 s onscreen (IQR: 3–20). However, the length of occurrences each year become shorter, from 10 s (IQR: 4–25) to 5 s (IQR: 2–13) between 2005 and 2015 (p<0.001). The median length of occurrences also declined by type of valence and impressions (Figure 2). Duration of positive for tobacco occurrences decreased over the years from 11 s (IQR: 5–26) in 2005 to 5 s (IQR: 2–14) in 2015 (p<0.001) (Figure 2A). Also, a clear decreasing trend in the median duration of exposure to explicit tobacco use occurrences from 18 s (IQR: 8–40) to 5 s (IQR: 2–15) (p<0.001), and tobacco brand appearances from 11 s (IQR: 5–20) to 2 s (IQR: 1–7) (p<0.001) was observed (Figure 2B).

The linear regression model showed that the number of total positive for tobacco occurrences in films did not significantly change in 2010 or in 2015 compared to 2005, although results pointed towards a decreasing trend. Similarly, no significant changes were observed in the number of explicit tobacco use and brand occurrences across the years. However, we observed that the number of total positive for tobacco and explicit tobacco use occurrences decreased by 48.7 (p=0.032) and 28.1 (p=0.039) in thriller films compared to dramas between 2010 and 2015. The number of positive for tobacco occurrences increased by 61.7 every five years since 2005 in films rated as ‘Not Recommended’ for audiences under 13 years of age compared to those rated to the ‘General Audience’ (p=0.036). Likewise, no significant changes were observed in the duration of positive impressions for tobacco occurrences (in minutes) across years nor in explicit tobacco use or brand occurrences (Table 1).

**Table 1:** Adjusted* multivariable linear regression models to assess the association between number of occurrences and duration of occurrences (minutes) with a positive valence for tobacco, impressions of explicit tobacco use and brands/logos in Spanish top-grossing movies and year, film genre and rating

*Variables*		*Positive for tobacco*		*Explicit tobacco use*		*Brands and logos*	
		*b (SE)*	*p*	*b (SE)*	*p*	*b (SE)*	*p*
**Number of occurrences**							
**Year**	2005 (Ref.)						
	2010	-2.64 (19.38)	0.893	8.36 (11.63)	0.479	-4.62 (2.57)	0.085
	2015	-18.07 (17.97)	0.325	2.29 (10.78)	0.834	-4.14 (2.38)	0.096
**Film genre**	Drama (Ref.)						
	Comedy	-2.25 (17.22)	0.897	-2.25 (10.33)	0.830	0.21 (2.29)	0.926
	Thriller	**-48.68 (21.33)**	0.032	**-28.08 (12.80)**	0.039	-2.56 (2.83)	0.374
**Rating**	General audience (Ref.)						
	Not recommended for audiences under 13 years of age	**61.73 (27.79)**	0.036	33.04 (16.68)	0.060	2.42 (3.69)	0.519
	Not recommended for audiences under 16 years of age	50.37 (30.51)	0.112	28.49 (18.31)	0.133	0.12 (4.05)	0.255
		R^2^=0.383	0.062	R^2^=0.293	0.196	R^2^=0.284	0.216
**Duration of occurrences** (minutes)							
**Year**	2005 (Ref.)						
	2010	-4.32 (6.12)	0.487	1.39 (4.03)	0.733	-2.28 (0.25)	0.254
	2015	-11.02 (5.54)	0.060	-5.11 (3.65)	0.177	-1.53 (1.45)	0.321
**Film genre**	Drama (Ref.)						
	Comedy	0.63 (5.20)	0.905	-0.52 (3.43)	0.881	0.78 (1.29)	0.562
	Thriller	-11.93 (7.09)	0.108	-8.48 (4.68)	0.084	1.61 (2.86)	0.588
**Rating**	General audience (Ref.)						
	Not recommended for audiences under 13 years of age	16.87 (8.49)	0.060	**12.44 (5.60)**	0.037	0.91 (2.86)	0.758
	Not recommended for audiences under 16 years of age	12.61 (9.57)	0.202	10.32 (6.31)	0.117	-1.07 (3.07)	0.737
		R^2^=0.376	0.095	R^2^=0.348	0.135	R^2^=0.369	0.609

Coefficients (b) adjusted for the variables in the table.

The multinomial logistic regression model fitted to understand changes in tobacco occurrence across year according to their valence and type of impression showed a decreased likelihood of observing a tobacco brand appearance (OR=0.32; 95% CI: 0.16–0.67; p<0.001) in 2010 compared to 2005 (Table 2). Similarly, we found decreased odds of observing a positive for tobacco occurrence (OR=0.34; 95% CI: 0.21–0.55) and increased odds of observing explicit tobacco use (OR=2.58; 95% CI: 1.53–4.33; p<0.001) in 2015 compared to 2005 (Table 2).

**Table 2:** Multinomial logistic adjusted* regression model of changes in tobacco occurrences by year under study according to the type of impression and their valence on the audience

*Year*		*AOR (95% CI)*	*p*
**2005**		Ref.	
**2010**	**Valence**		
	Other (Ref.)	1	
	Positive for tobacco	0.80 (0.47–1.37)	0.415
	**Type of occurrence**		
	Implied tobacco use (Ref.)	1	
	Explicit tobacco use	1.32 (0.83–2.09)	0.236
	Tobacco products paraphernalia	0.76 (0.49–1.20)	0.238
	Tobacco logo or brand appearances	**0.32 (0.16–0.67)**	<0.001
**2015**	**Valence**		
	Other (Ref.)	1	
	Positive for tobacco	**0.34 (0.21–0.55)**	<0.001
	**Type of occurrence**		
	Implied tobacco use (Ref.)	1	
	Explicit tobacco use	**2.58 (1.53–4.33)**	<0.001
	Tobacco products paraphernalia	1.12 (0.67–1.86)	0.671
	Tobacco logo or brand appearances	0.93 (0.47–1.87)	0.847
		LRS	
		χ^2^=65.72	<0.001

* AOR: adjusted odds ratio; adjusted for the variables in the table. LRS: likelihood ratio statistic.

### Population exposure to tobacco impressions

Reach of positive for tobacco occurrences in Spanish top-grossing films also decreased over the years from 499.5 million (95% CI: 265.2–733.7) in 2005 to 245.0 million (95% CI: 92.8–397.2) in 2015 tobacco impressions (Table 3). The rate of exposure to impressions with a positive valence for tobacco per minute of film significantly decreased from 15.9 (95% CI: 15.86–15.86) per 1000 spectators in 2005 to 8.7 (95% CI: 8.67–8.67) in 2010, and 3.5 (95% CI: 3.54–3.54) in 2015. An inverse trend, however, was observed for those impressions with a neutral or negative for tobacco valence since its exposure rate increased between 2005 and 2010 from 0.5 (95% CI: 0.53–0.53) to 1.0 (95% CI: 0.96–0.96), and decreased between 2010 and 2015 to 0.8 (95% CI: 0.82–0.82). No statistically significant differences were observed.

**Table 3:** Estimated gross tobacco occurrences delivered (impressions) in 30 sampled films per year in Spain in 2005, 2010 and 2015 containing tobacco imagery

*Release year in Spain*	*Gross tobacco impressions (million)*			
	*Total number of occurrences*		*Positive for tobacco occurrences*	
	*Sum (95% CI)*	*Duration (min)*	*Sum (95% CI)*	*Duration (min)*
**2005**	534.80 (301.22–768.37)	161.42	499.45 (265.21–733.69)	156.18
**2010**	228.15 (76.87–379.42)	57.77	214.99 (62.66–367.33)	52.03
**2015**	315.02 (117.20–512.83)	51.80	244.99 (92.76–397.22)	42.11
**Total**	1077.96 (745.79–1410.12)	270.98	959.43 (646.08–1272.79)	250.32

### Smokers’ depiction in films

We observed a change of pattern in the type of role most smokers played on screen from a leading role in 2005 to extras in 2010 and a supporting role in 2015 (p<0.001). A similar statistically significant relationship was also observed by sex, age, type of venue, and social context of smoking. As shown in Table 4, the proportion of smokers onscreen in 2005 was higher in men characters, while in 2015 it was higher in women (79.0% vs 54.9%; p<0.001). For age, the proportion of smokers that seemed to be aged ≥30 years of the total smokers onscreen was the highest in 2005, increasing in predominance even more in 2015 (p=0.004). Although the proportion of characters that seem minors was never too high, it decreased with time and in 2015 we did not detect any. For the type of smoking policy environment, we found an increase in the proportion of explicit tobacco use occurrences taking place in public workplaces (8.2% vs 66.4%), but a decreasing trend for private spaces (27.0% vs 15.1%), public hospitality venues (43.4% vs 8.9%) and other public spaces (21.4% vs 9.6%) in 2015 compared to 2005, and 2005 and 2010, respectively. Finally, for social context, there was a decrease in the proportion of onscreen smoking for social acceptance from 2005 to 2015. However, identity smoking increased in 2010 (3.3% vs 10.9%) and 2015 (3.3% vs 11.5%) compared to 2005, ritual smoking decreased in 2010 compared to 2005 (56.1% vs 70.5%), while pleasure smoking decreased in 2015 compared to 2005 (15.0% vs 6.8%) (Table 4).

**Table 4:** Variables associated with explicit tobacco use occurrences in 30 Spanish films by premiere year

*Variables*	*Categories*	*Total*		*2005*		*2010*		*2015*		
		*n*	*%*	*n*	*%*	*n*	*%*	*n*	*%*	*p*
	**Total**	601	100	224	37.3	184	30.6	193	32.1	
**Part**	Leading	180	30.0	99	44.2	52	28.3	29	15.0	**<0.001**
	Supporting	199	33.1	51	22.8	42	22.8	106	54.9	
	Extra	222	36.9	74	33.0	90	48.9	58	30.1	
**Sex**	Male	405	67.4	177	79.0	141	76.6	87	45.1	**<0.001**
	Female	196	32.6	47	21.0	43	23.4	106	54.9	
**Age** (years)	Minors (<18)	19	3.2	15	6.8	4	2.2	0	0.0	**<0.001**
	Young adults (18–30)	137	23.0	64	28.8	44	23.9	29	15.0	
	Other adults (>30)	445	74.0	145	64.7	136	73.9	164	85.0	
**Tobacco product**	Manufactured cigarettes	523	87.0	190	84.8	161	87.5	172	89.1	0.448
	RYO cigarettes	35	5.8	13	5.8	13	7.1	9	4.7	
	Other	43	7.2	21	9.4	10	5.4	12	6.2	
**Environment**	Closed	397	66.2	144	64.6	119	64.7	134	69.4	0.294
	Semi-opened	35	5.8	14	6.3	7	3.8	14	7.3	
	Opened	168	28.0	65	29.1	58	31.5	45	23.3	
**Venue**	Private spaces^a^	95	22.0	43	27.0	30	23.6	22	15.1	**<0.001**
	Public workplace	111	25.7	13	8.2	1	0.8	97	66.4	
	Public hospitality venues^b^	134	31.0	69	43.4	52	40.9	13	8.9	
	Other public spaces^c^	92	21.3	34	21.4	44	34.7	14	9.6	
**Social context of smoking**	Defiance	13	2.2	4	1.9	3	1.6	6	3.1	**<0.001**
	Social	80	13.6	45	21.0	12	6.6	23	12.0	
	Policy environment	10	1.7	6	2.8	3	1.6	1	0.5	
	Identity	49	8.3	7	3.3	20	10.9	22	11.5	
	Ritual	376	63.8	120	56.1	129	70.5	127	66.1	
	Pleasure or comfort	61	10.4	32	15.0	16	8.7	13	6.8	

RYO: roll-your-own.

a Private spaces: homes (n=87), private transport (n=8).

b Public hospitality venues: bars and restaurants (n=120), nightlife venues (n=14).

c Other public spaces: hotels (n=12), stations and airports (n=7), schools (n=1), healthcare centers (n=4), governmental buildings (n=1), public transport (n=6), theatres (n=19), concert venues (n=10), cinemas (n=4), dancing school (n=1).

## DISCUSSION

Our findings suggest that positive for tobacco occurrences in films did not increase in Spanish top-grossing films after a comprehensive tobacco control policy completely banning tobacco marketing communication was implemented in Spain. Instead, we observed a decreasing trend in the number and duration of tobacco occurrences, and in the amount of tobacco impressions received by the audience, suggesting that banning all commercial communication on tobacco products, including product placement in media (Law 7/2010), may have had an impact in reducing tobacco imagery in films. Nonetheless, our results highlight a change in how smokers are depicted onscreen and where smoking takes place across years underlying a transition in how smoking is marketed in Spanish top-grossing films.

Millet and Glantz^3^ suggested that the relative importance of smoking in films will be higher as countries implement more stringent restrictions on tobacco marketing communication, since smoking in films would become the world’s largest vector for sustaining the smoking appeal and promote its initiation^14^. Instead, according to our results, tobacco appearances did not increase after a complete ban of tobacco advertising was implemented in Spain, but quite the opposite. However, exposure to tobacco imagery in films is still considerably high impacting, producing 245 million impressions positive for tobacco on Spanish audiences in cinemas. These exposure levels highlight that, even if tobacco imagery in films is decreasing, it continues to be pervasive and an open-door for promoting smoking in those countries where tobacco products marketing communication is completely banned.

Moreover, most of the observed tobacco occurrences were described as positive for tobacco, ranging from 93.0% in 2005 to 88.0% in 2015 of total observed. These findings indicate that portrayals of smokers in films largely ignore the negative consequences of smoking. Previous studies have suggested that tobacco imagery in films hardly ever portrays health costs and exaggerate levels of smoking by up to four times, contributing to heightened estimates of prevalence^14^. Hence, our results should encourage policymakers and stakeholders to advocate for a more realistic depiction of tobacco health hazards onscreen since media plays a key role in shaping the public’s perceptions of who is responsible for public health problems and their solutions, thus ‘framing’ issues for the public^15^.

Smoking is portrayed in all film rating categories; however, it is most prevalent in ‘Not recommended for audiences under 13 years of age rated films, exposing teenagers and young adults to smoking onscreen. Smoking in films is a potent stimulus for youth smoking^3^ that accounts for one-third to one-half of adolescent smoking onset^16^. Our results are aligned with a previous study in the UK that found tobacco appearances in 70% of films rated as suitable for children under 15 years of age^17^. This highlights that, despite the WHO recommendation to assign an ‘Adult’ rating to all films with tobacco content^1^ and the robust evidence that R-rating restrictions prevent onset of tobacco use^14,16^, film classification bodies or governments have not yet implemented it. Instead, many governments certify films with tobacco use as appropriate for youth by providing generous subsidies to the film industries, since no distinction is made between projects whose content plays an important role in recruiting adolescents to smoke and those that do not^18^.

Moreover, the unexpected proportion of people exposed to positive for tobacco impressions in the top-grossing films in Spain is of importance. Even more so, since exposure rate to positive for tobacco impressions in Spain is unfortunately underestimated in our study as we were only able to measure exposure in cinemas but not through other means such as streaming platforms. Also, the magnitude of such underestimation of the exposure to tobacco imagery may have increased in each time-point as on-demand and streaming services have not ceased to gain in popularity since they were introduced in the market while cinema audiences show a decreasing trend (2005: 143.9 million spectator vs 2010: 101.6 million, and 2015: 96.1 million)^10^. The association of this decrease with the implementation of the smoke-free legislation is appealing, but it is a too simplistic and unlikely explanation, since films are not mirroring current society, but the society in different calendar times, with different social norms at each time.

Accordingly, the Spanish government, to comply with Article 13, paragraph 45, of the WHO FCTC, should ensure that the existing comprehensive ban on TAPS also covers films and TV series by making this ban explicit. Moreover, as suggested by the WHO^1^, they should move towards smoke-free films by forcing production companies to certify no pay-offs by the tobacco industry; require strong anti-smoking advertisements; require adult ratings for all films with tobacco imagery; and make media productions with smoking ineligible for public subsidies.

Finally, we observed that onscreen smokers shifted from leading men roles to supporting women roles from 2005 to 2015. These changes could be explained by how smoking prevalence distribution has evolved over time by sex and age as the prevalence of smoking in men has decreased drastically, while in women has declined less significantly^4^. However, we conducted a cross-sectional study and, therefore, we cannot rule out the hypothesis that the change in smoking prevalence in Spain has not been interfered by tobacco industry integrated marketing communications campaigns or strategies over the years, excluded from the study.

### Strengths and limitations

This study has some limitations. First, our analysis is limited to three years (2005, 2010, 2015) overlooking what has happened in between over the 15 years’ period. However, these time points in calendar time can well indicate secular changes related to differences in the legislation in place at the time. Second, we coded a relatively small sample of 10 films for each year. We decided to include those with the highest-grossing titles, i.e. those seen by more people at movie theaters. Films with lower public profiles might have different tobacco content; however, top-grossing films reach larger audiences and consequently are a more accurate proxy for population exposure to tobacco imagery in films. Third, as our study only focused on Spanish films, we excluded films produced in other countries, including Hollywood ones, that have the highest audience reach worldwide and in Spain. However, we believe that films produced in Spain are those that can fully reflect how the tobacco industry reacts to the implementation of tobacco control policies domestically. Future research can be focused on all films distributed in Spain to understand if trends are different depending on the country of production. Fourth, despite the potential limitations of the data collection method used, we applied the same methodology that previous studies on tobacco imagery on films and TV^12,13,17^. Films were viewed by two trained observers. Disagreements were solved by consensus with a third viewer, reducing the risk of bias. Fifth, our study provides a valid picture of the smoking-related occurrences in films but under ‘controlled conditions’ since the viewers were able to pause the film, rewind and watch the scene again, whereas spectators in cinemas cannot pause and rewind. Therefore, people’s perceptions in cinemas could underestimate the real number of occurrences. Exploring people’s perceptions of tobacco occurrences just after viewing a film (in real conditions) against the actual number of occurrences should be explored in future studies to better characterize the exposure to tobacco imagery in movies. Sixth, in our study, the proportion of occurrences depicting RYO tobacco smoking might be overestimated since we could not distinguish them from the smoking marijuana joints. However, joints may mix marijuana with tobacco leaf and, in any case, they represented a low proportion (<6%) of the total number of explicit tobacco-use occurrences. Finally, our findings could be underestimating the impression of tobacco occurrences in the films analyzed as we did not have viewing rates of these films through on-demand or streaming services nor through TV broadcasting. In spite of the increase in on-demand services by the youngest Spaniards, linear television is still important in this group. In 2020, both watching linear TV daily was very common among teenagers 13 to 17 years old (63%) concurrently with watching video/TV on demand every day (69%)^19^.

Our study has also some strengths as it is, to the best of our knowledge, the first to assess changes in film smoking before and after the implementation of a complete ban on tobacco products marketing communications in the European Union and second worldwide; and the first to describe how smoking depiction onscreen has evolved to target new segments of the population over the years.

## CONCLUSIONS

In Spain, the implementation of a complete ban on tobacco products marketing communication, including product placement in media, was followed by a decrease in tobacco incidents across top-grossing Spanish films. Yet, exposure to smoking in films is still unacceptably high, especially in films rated as not suitable for audiences under 13 years of age, exposing audiences to pervasive indirect tobacco advertising. Hence, our findings should encourage the Spanish government to make TAPS bans in films and TV explicit to stringent existing regulations; to require certification of no pay-offs by the tobacco industry and strong anti-smoking advertisements; to require adult ratings for all films with tobacco imagery; and to make media productions with smoking ineligible for public subsidies to decrease Spanish population exposure to tobacco imagery. Closing the actual open-door to tobacco marketing communications through cinema would be a strong step-forward for a smoke-free generation in Spain since these interventions have proven to be effective to reduce smoking uptake and recurrences in adolescents^1,14,16^.

## Data Availability

The data supporting this research are available from the authors on reasonable request.
